# Editorial: The role of minerals and trace elements in chronic diseases

**DOI:** 10.3389/fcell.2023.1333011

**Published:** 2023-11-21

**Authors:** Muhammad N. Aslam, Shane M. Heffernan, James Varani

**Affiliations:** ^1^ Department of Pathology, The University of Michigan Medical School, Ann Arbor, MI, United States; ^2^ Faculty of Science and Engineering, Swansea University, Swansea, Wales, United Kingdom

**Keywords:** minerals, trace elements, calcium, chronic diseases, prevention and control, cellular processes, molecular processes

Several disease conditions that collectively can be described as chronic and long-latency are becoming major public health issues in Western society. Certain cancers, heart and cardiovascular diseases, metabolic diseases (e.g., type II diabetes and fatty liver disease), progressive bone loss and even certain neurodegenerative diseases have always been associated with the aging process. These chronic diseases are increasing as a result of the population aging. More problematic, these same conditions (metabolic disorders and cancers) are showing up in younger individuals. What is triggering this trend? Efforts to mitigate diseases that are chronic, and long-latency focus largely on managing symptoms rather than cure.

It has long been known that dietary micronutrients–especially inorganic minerals and the vitamins and hormones needed for their effective utilization–are critical to staving off the consequences of common chronic ailments. We are well aware, of course, that an adequate calcium intake is critical to all aspects of health and well-being (Peterlik and Cross, 2009). Calcium as a colon cancer chemopreventive is accepted (Keum et al., 2014) and we have mechanistic insight as to the many ways that calcium might function to reduce colon polyp formation (Aslam et al., 2012; Singh et al., 2015; Swaminath et al., 2019). The importance of minerals such as magnesium, iron, copper and zinc is known because deficiencies in intake do exist and are associated with susceptibility to specific ailments (Hans and Jana, 2018; Lennie et al., 2018). Beyond this, however, our understanding of how most trace elements participate in disease prevention is limited. Trace element participation in specific biochemical processes may be known, but how these processes—individually or collectively—affect overall health is unclear. What constitutes optimal (or even minimally required) levels of such trace elements is not known. It is also important to note that many trace elements can be toxic if the level of intake is too high. Equally important to note is that trace elements can overlap in how they affect critical biological processes. For example, calcium-binding sites on critical regulatory proteins are, in fact, cationic metal binding sites (Huang et al., 2009; Carrillo-Lopez et al., 2010). Thus, relative amounts of the potentially competitive elements present in the milieu and their relative affinities for specific binding sites have an impact on protein function and, ultimately, determine biological activity. Effects on overall health and chronic disease prevention is the result.

Impactful research into the mechanisms by which trace elements promote or inhibit chronic disease prevention is challenging. In addition to the issues cited above, chronic disease prevention is difficult because it requires precise cellular and molecular investigation in conjunction with the use of sophisticated animal and/or human tissue models that can (hopefully) act as surrogates for the chronic diseases we are trying to prevent. More research is needed!

This Research Topic of Frontiers in Cell and Developmental Biology (Molecular and Cellular Pathology section) hopes to encourage such research. The following published studies under this topic have discussed issues related to mineral overload or under-use of some of these minerals individually or in combination.

Iron overload is a condition associated with an increased prevalence of osteoarthritis (OA) in the elderly population, but the exact role of iron in OA development is not yet established. Jing et al. aimed to explore the connection between iron overload and OA. The results showed that iron-overloaded mice had increased iron accumulation, dysregulated iron transport and greater cartilage destruction. Inhibition of divalent metal transporter-1 (DMT1), a pivotal iron transporter, suppressed the inflammatory response and extracellular matrix (ECM) degradation, suggesting that it could be an attractive new target for OA treatment.

Cai et al. reviewed the findings and observations in the field of iron overload-related OA. The cellular and molecular mechanisms associated with iron overload and the negative influence that iron overload has on joint homeostasis were elucidated. Interrupting the pathologic effects of iron overload is discussed as a promising path for the development of improved therapeutics in the field of OA.

Boneski et al. investigated age-dependent changes in the vertebral bone and intervertebral disc using the Abcc6^−/−^ mouse model of pseudoxanthoma elasticum (PXE), a progressive human metabolic disorder characterized by mineralization of the skin and elastic tissues. The results showed that loss of ATP-binding cassette sub-family C member 6 (ABCC6) compromises vertebral bone quality and dysregulates osteoblast-osteoclast coupling, while the treatment with oral potassium citrate inhibited the osteoclastic response and improved mechanical properties of the bone.

In the last published study, our group Varani et al. assessed the effects of a red marine algae-derived multi-mineral intervention (known as Aquamin^®^) on protein expression patterns in human colon organoids (derived from healthy human colonic tissue). For this study, organoids were maintained under control conditions or treated with a combination of bacterial lipopolysaccharide and three pro-inflammatory cytokines to simulate inflammatory bowel disease in an *ex vivo* setting. The pro-inflammatory stimulus altered the expression of proteins that influence innate immunity and promote inflammation. Among these were multiple human leukocyte antigens (HLA) [e.g., HLA-DPA1, HLA-DPB1, HLA-DRA, HLA-DRB isoforms 1, 3, 4 and 5, HLA-B, HLA-E, HLA-F and HLA-DMB]. Additional upregulated proteins included membrane-associated phospholipase A2 (PLA2G2A), Indoleamine 2,3-dioxygenase (IDO1) and Tryptophan-tRNA ligase, cytoplasmic (WARS). When the mineral supplement was included along with the same pro-inflammatory stimulus, levels of these same proteins were reduced compared to levels obtained in the absence of the mineral supplement. Mineral supplementation also reduced the expression of other proteins (FGB, FGG and SPARC) that promote inflammation and increased expression levels of proteins (FBP2, SLC11A2, SIRT3, S100A7, PBLD, HPGD, GSTA1 and DCD) with anti-inflammatory and antioxidant properties. The same mineral product also upregulated proteins that contribute to gut barrier formation and tissue strength by improving cell-cell/cell-matrix adhesions. Among these were multiple keratins and mucins, filaggrin, tight junction protein-1 (TJP1), cadherins [CDH-1, -3 and -17], desmosomal and hemidesmosomal proteins [DSC2, DSG2, DSP, JUP], trefoil protein-2 (TFF2) and multiple CEACAMs. Improved barrier function assessed by transepithelial electrical resistance (TEER) and tissue cohesion was also observed with the same mineral supplement under both control conditions and in the presence of the pro-inflammatory stimulus. These data validated previous findings from human colon organoid studies and a biomarker trial in human subjects (Attili et al., 2019; Aslam et al., 2020; McClintock et al., 2020; Aslam et al., 2021). Overall, the data suggest that improving barrier structure and function may have implications regarding inflammatory bowel disease prevention. These *in vitro* studies were conducted in parallel with an ongoing trial (ClinicalTrials.gov ID - NCT03869905) interrogating the role of the same mineral combination (Aquamin^®^) in subjects with mild to moderate ulcerative colitis (UC). Given this data, we anticipate that the ongoing clinical trial will significantly contribute to confirming the potential benefits of this approach in patients with UC.

In conclusion, the studies presented in this special edition offer significant potential to interrupt the pathologic effects of mineral imbalance. These findings may lead to new therapeutic targets and, ultimately, improve ways to prevent chronic, long-latency diseases or mitigate their consequences.
